# Transcriptome Analysis Reveals the Immunosuppression in Tiger Pufferfish (*Takifugu rubripes*) under *Cryptocaryon irritans* Infection

**DOI:** 10.3390/ani14142058

**Published:** 2024-07-13

**Authors:** Yong Chi, Robert Mukiibi, Hongxiang Zhang, Haien Zhang, Weidong Li, Diego Robledo, Songlin Chen, Yangzhen Li

**Affiliations:** 1 State Key Laboratory of Mariculture Biobreeding and Sustainable Goods, Yellow Sea Fisheries Research Institute, Chinese Academy of Fishery Sciences, Qingdao 266071, China; chiyong4783@foxmail.com; 2 The Roslin Institute and Royal (Dick) School of Veterinary Studies, The University of Edinburgh, Edinburgh EH25 9RG, UK; rmukiibi@exseed.ed.ac.uk (R.M.); diego.robledo@roslin.ed.ac.uk (D.R.); 3 Tangshan Haidu Seafood Co., Ltd., Tangshan 063506, China; 15954672795@163.com (H.Z.); 18804895712@163.com (H.Z.); weidong1970@hotmail.com (W.L.); 4 Fishery Research Institute, Tangshan Academy of Agricultural Sciences, Tangshan 063001, China; 5 Department of Genetics, Universidade de Santiago de Compostela, 15706 Santiago de Compostela, Spain

**Keywords:** fugu, RNA-seq, immune response, parasite, aquaculture

## Abstract

**Simple Summary:**

Parasite diseases are recognized as major concerns in the fugu aquaculture industry in China. While understanding the genetic mechanisms of immune response is a crucial step which can facilitate disease control and selective breeding. In this study, transcriptome analysis was performed to identify key genes and understand the underlying mechanisms associated with *Cryptocaryon irritans* resistance. Finally, our study provided insights into the immunosuppression in fugu under *C. irritans* infection.

**Abstract:**

The tiger pufferfish (*Takifugu rubripes*), also known as fugu, has recently suffered from severe *C. irritans* infections under aquaculture environment, yet the underlying immune mechanisms against the parasite remain poorly understood. In this study, we conducted a comprehensive transcriptome analysis of the gill tissue from infected and uninfected fish using PacBio long-read (one pooled sample each for seriously infected and healthy individuals, respectively) and Illumina short-read (three pools for mildly infected, seriously infected, and healthy individuals, respectively) RNA sequencing technologies. After aligning sequence data to fugu’s reference genome, 47,307 and 34,413 known full-length transcripts were identified and profiled in healthy and infected fish, respectively. Similarly, we identified and profiled 1126 and 803 novel genes that were obtained from healthy and infected fish, respectively. Interestingly, we found a decrease in the number of alternative splicing (AS) events and long non-coding RNAs (lncRNAs) after infection with *C. irritans*, suggesting that they may be involved in the regulation of the immune response in fugu. There were 687 and 1535 differentially expressed genes (DEGs) in moderately and heavily infected fish, respectively, compared to uninfected fish. Kyoto Encyclopedia of Genes and Genomes (KEGG) analyses showed that immune-related DEGs in the two comparison groups were mainly enriched in cytokine-cytokine receptor interactions, ECM-receptor interactions, T-cell receptor signaling pathways, Th1 and Th2 cell differentiation, and Th17 cell differentiation pathways. Further analysis revealed that a large number of immune-related genes were downregulated in infected fish relative to uninfected ones, such as CCR7, IL7R, TNFRSF21, CD4, COL2A1, FOXP3B, and ITGA8. Our study suggests that *C. irritans* is potentially a highly efficient parasite that may disrupt the defense mechanisms of fugu against it. In addition, in combination of short-read RNA sequencing and previous genome-wide association analyses, we identified five key genes (NDUFB6, PRELID1, SMOX, SLC25A4, and DENND1B) that might be closely associated with *C. irritans* resistance. This study not only provides valuable resources of novel genic transcripts for further research, but also provides new insights into the immune mechanisms underlying *C. irritans* infection response in farmed fugu.

## 1. Introduction

As the demand for sustainable aquaculture continues to grow, various marine species are gaining attention for their economic and nutritional benefits. The tiger pufferfish (*Takifugu rubripes*), also known as fugu, is widely distributed in the coastal areas of China, Japan, and Korean, and has become an important economic marine fish because of its delicious flesh and high nutritional value [1,2]. It is becoming a promising industry due to the decrease in wild resources and the increase in artificial culture [3]. In recent years, both the growing intensive aquaculture production of fugu and the high water temperatures in the summertime have provided conditions for both pathogenic and parasitic infection development which are causing significant economic losses to the farming industry. One of the most important parasitic infections is cryptosporidiosis, caused by a parasite *C. irritans* which mainly invades the gills, fins, and skin of fish and forms white spots on the surface [4,5]. Infection by *C. irritans* causes suffocation, osmotic imbalance, and mass mortality of the fish [6]. Currently, there is no effective method for eradicating cryptosporidiosis. Therefore, it is critical to study the immune mechanisms of fugu in response to *C. irritans* infection, as these could support the development of genetic tools and strategies to combat the detrimental impacts of the disease to the fugu farming industry.

In the recent past, several studies have been undertaken to understand fugu immune response to *C. irritan* infection as well as molecular pathogenesis of the parasite [5,7,8]. Transcriptome sequencing provides a viable high-throughput analytical method for discovering the transcriptional expression status of genes, with significant advantages in revealing the regulatory networks and molecular mechanisms of the tissue under study [9,10]. Currently, most transcriptome studies are performed with second-generation sequencing, which have been widely used to study the molecular mechanisms of infection with *C. irritan* in different aquaculture species [11,12,13,14,15,16,17,18].

Meanwhile, third-generation sequencing technologies show unparalleled advantages over second-generation sequencing technologies, such as the ability to generate long reads and thus facilitating to analyze splicing isoforms for improved genome annotation [19,20,21]. Full-length transcriptome-based annotation of genomic data can add some new genetic information [22]. Recently, PacBio-based transcriptome analyses have been reported in a variety of aquaculture species [23,24,25]. However, to date, no full-length transcriptome study has been performed in fugu. 

Here, we performed a comprehensive transcriptome analysis between healthy and *C. irritant*-infected fish using PacBio RNA sequencing and second-generation short-read RNA sequencing. We explored the molecular responses of fugu after infection with *C. irritans* using Illumina short-read sequencing, providing valuable data to elucidate the immune mechanisms after *C. irritans* infection. In addition, we constructed full-length transcripts and analyzed their structure and function for the first time in fugu, which further enriched the gene resources of this species.

## 2. Materials and Methods

### 2.1. Sample Collection

During the summertime of 2022, we observed that fish from one of the aquaculture ponds almost lost their appetite, and further pathological examinations confirmed the infection by *C. irritans*. Immediately, gill samples of mildly and seriously ill individuals were collected. We assessed the severity of infection by counting the number of white spots on the skin to ensure consistent and accurate criteria across individuals, allowing for a standardized and objective measure of infection severity. The number of white spots in a square centimeter of the skin was utilized to define the level of infection with individuals having 3–6 spots defined as the mild group (MG) while those with 10 and more white spots were defined as the seriously infected group (SG). Fish in the healthy group (HG) were obtained in different ponds and were free of any signs and symptoms of cryptosporidiosis infection. In total, 27 fish were used in this study which included nine individuals from each group. Before sampling, the fish were euthanized by immersion in a tricaine methanesulfonate (MS-222) solution (20 mg/L). Gill tissue was then extracted from each euthanized fish and flash-frozen in liquid nitrogen then stored in an ultra-low temperature freezer (−80 °C) until use. 

### 2.2. RNA Isolation, Library Construction and Sequencing

Total RNA was extracted from the gill tissue by the Trizol reagent kit (Invitrogen, Carlsbad, CA, USA) according to the manufacturer’s instructions. mRNA was enriched by Oligo (dT) magnetic beads. In each group, equal amounts mRNA (0.3 μg) of 3 individuals were then randomly pooled into one, and sample names were recorded as HG-1, HG-2, HG-3, MG-1, MG-2, MG-3, SG-1, SG-2, and SG-3. For the short-read RNA-seq, cDNA libraries were prepared using TruSeq Stranded mRNA LT Sample Prep Kit (Illumina, San Diego, CA, USA), followed by purification and size selection. The cDNA libraries were subjected to the Illumina Novaseq6000 platform for sequencing (services provided by the Gene Denovo Biotechnology Co., Ltd., Guangzhou, China). For PacBio library construction, the three mRNA pools of the HG and SG were further pooled into one sample separately and named FL-HG (full-length healthy gill) and FL-DG (full-length diseased gill), respectively. Then, two pooled mRNA samples were reverse transcribed into cDNA using a Clontech SMARTer PCR cDNA Synthesis Kit (Takara Bio, San Jose, CA, USA), followed by fragment size selection (>4 Kb) on the BluePippin™ Size-Selection System (Beverly, MA, USA). Then, large-scale PCR was performed for SMRTbell library construction. Next, cDNAs were DNA damage repaired, end repaired, and sequencing adapters were subsequently ligated to cDNA. The SMRTbell templates with sequencing primers were annealed and combined with polymerase, and sequencing was performed on the PacBio Sequel platform (services provided by Gene Denovo Biotechnology Co., Ltd., Guangzhou, China). 

### 2.3. Differential Expression Analysis and Functional Enrichment

For short-reads RNA-seq data, raw reads were filtered using the software fastp v0.23.4 [26]. The filtration criteria are as follows: (1) remove reads containing an adapter, (2) remove reads with an N ratio greater than 10%, (3) remove reads with all A bases, (4) remove low-quality reads (the number of bases with a quality value Q ≤ 20 accounts for more than 50% of the whole read). Clean reads were then mapped to the reference genome (version: fTakRub1.3) using HISAT2 v2.0.4 [27]. Stringtie v2.21 [28] was used to assemble gene transcripts from the alignments in conjunction with genome annotation files and subsequently quantify the expression of these transcripts by counting the reads overlapping the assembled transcripts in each sample. Differential gene expression between healthy fish and infected fish was performed (i.e., HG vs. MG and HG vs. SG) using the Bioconductor R package DESeq2 [29]. Genes with a false discovery rate (FDR) below 0.05 and absolute fold change ≥ 2 were considered as differentially expressed genes (DEGs). Function enrichment analysis of the DE genes was performed through GO term and KEGG pathway enrichment analyses. In addition, we performed gene set enrichment analysis (GSEA) using expression information for all genes to determine differences in gene expression in healthy and infected fish [30]. 

### 2.4. Key DEGs Screening and Protein–Protein Interaction (PPI) Network

Based on the results of enrichment analysis, we screened DEGs in immune-related pathways. Subsequently, we screened key genes that were differentially expressed in both comparison groups. To understand the potential relevance of the key genes in the two comparative groups, the online software STRING (https://string-db.org/, accessed on 4 June 2024) was used to compare the selected two groups of common immune-associated DEGs with the STRING database, while protein interactions of reference species were used to construct STRING PPI networks. The degree value for each gene was calculated by the software Cytoscope (v3.10.2), with default parameters listed from largest to smallest [31].

### 2.5. Comparison of DEGs and Candidate Genes in QTL Regions

All DEGs obtained from the HG vs. MG and HG vs. SG groups were compared with the candidate genes in QTL regions from our previous genome-wide association studies (GWAS) of *C. irritans* resistance [32]; the overlapping genes were considered as key genes which would further provide new insights into the genetic mechanisms of disease resistance.

### 2.6. Processing of PacBio Full-Length Sequencing Data

We processed PacBio raw reads using SMRTlink 8.0 software to obtain high-quality sequences. We obtained the cyclic consensus sequence (CCS) from the subread BAM file with the parameters set to: min predicted accuracy = 0.8, min length = 50, max length = 15,000, min z score = −9999, min passes = 1, max drop fraction = 0.8, min read score = 0.65, and polish = false. The full-length non-chimeric (FLNC) reads were obtained by removing primers, barcodes, polyA tail trimming, and concatemer of full passes. To obtain the consistency sequence, similar FLNC reads were clustered hierarchically using minimap2 [33]. The consistent sequences were then further corrected using the Quiver algorithm to obtain high-quality (HQ) isoforms. The HQ isoforms were then mapped to reference genome (version: fTakRub1.3) using minimap2. The minimap2 output bam format files and genome annotation files were used to identify genes and transcripts. Consistent sequences aligned to unannotated regions of a gene are considered isoforms of novel genes, and consistent sequences aligned to different exons of a known isoform are considered novel isoforms of known genes.

### 2.7. Isoforms Annotation and Structure Analysis

To understand the function of the new isoforms, the new isoforms were compared with the NCBI non-redundant protein (Nr) database, the Swiss-Prot protein database and the Kyoto Encyclopedia of Genes and Genomes (KEGG) database. AS events were identified using the SUPPA tool [34] and seven major AS events were categorized and counted. The protein-coding potential of new isoforms were assessed using two software, CPC [35] and CNCI (https://github.com/www-bioinfo-org/CNCI, accessed on 4 June 2024) [36]. Isoforms were also mapped to the Swiss-Prot database and the intersection of both non-protein annotation results and non-protein coding potential results were selected for lncRNA analysis.

## 3. Results

### 3.1. Overview of the PacBio Sequencing Data

**Figure 1:**
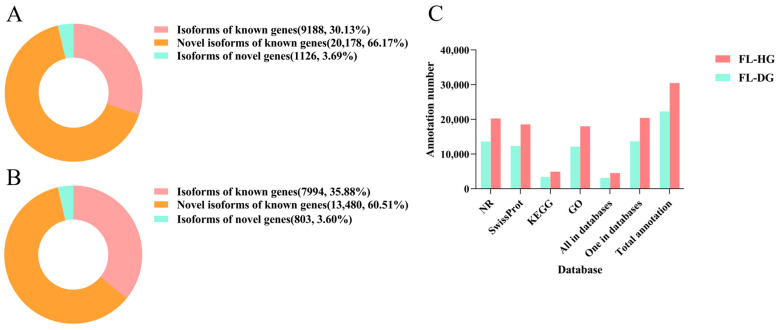
Characteristics of transcripts and novel gene annotation. (**A**) Classification statistics of full-length transcripts in the FL-HG. (**B**) Classification statistics of full-length transcripts in the FL-DG. (**C**) Statistic results of the NR database, Swiss–Prot protein database, KEGG database, and GO database. The abscissa represents different databases, and the ordinate represents the number of annotations.

In this study, two PacBio full-length transcriptome profiles (FL-HG and FL-DG) of *T. rubripes* were generated based on pooled mRNA from nine healthy and severely infected individuals. Detailed statistics of PacBio sequencing results were shown in Table 1. Based on the self-corrected subreads, 786,806 and 609,313 CCS reads were obtained in the FL-HG and FL-DG, respectively (Table 1). Subsequently, 47,307 and 34,413 HQ isoforms were identified from the FL-HG and FL-DG, respectively. Upon read alignment with the fugu genome, 35,931 (75.95%), 385 (0.81%), and 10,991 (23.23%) unique, multiple, and unmapped reads were identified in the FL-HG, and 25,614 (74.43%), 271 (0.79%), and 8528 (24.78%) reads were identified in the FL-DG (Table 1). Classification of the full-length transcripts showed that, in the FL-HG, there were 9188 (30.13%), 20,178 (66.17%), and 1126 (3.69%) isoforms of known genes, novel isoforms of known genes, and isoforms of novel genes, respectively (Figure 1A). Similarly, the information of isoforms of known genes, novel isoforms of known genes, and isoforms of novel genes in the FL-DGs were 7994 (35.88%), 13,480 (60.51%), and 803 (3.60%), respectively (Figure 1B).

### 3.2. Functional Annotation of the Transcripts

In the FL-HG, 20,403 transcripts were annotated in at least one database and 4517 transcripts were annotated in all databases (NR, Swiss-Prot, GO, and KEGG). In total, 20,242, 18,555, 18,027, and 4905 transcripts were annotated in the NR, Swiss-Prot, GO, and KEGG databases, respectively (Figure 1C). In the FL-DG, 13,696 transcripts were annotated in at least one database and 3101 transcripts were annotated in all databases; 13,572, 12,317, 12,100, and 3399 transcripts were annotated from the NR, Swiss-Prot, GO, and KEGG databases, respectively (Figure 1C).

### 3.3. Alternative Splicing (AS) and lncRNA Analysis

**Figure 2:**
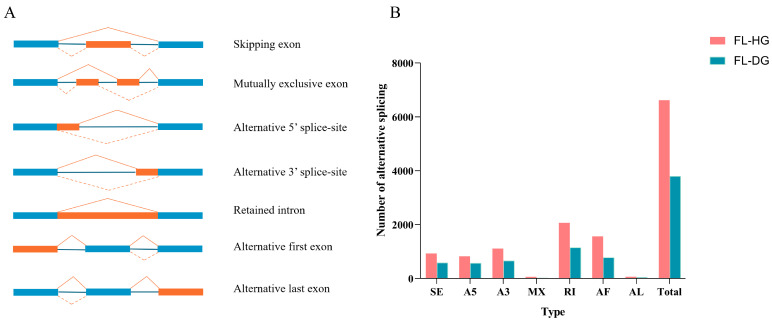
Alternative splicing (AS) analysis based on the full-length transcripts. (**A**) Seven types of AS forms. Blue blocks represent constitutive exons, orange blocks represent alternatively spliced exons. (**B**) Statistic results of AS events: SE (skipping exon), MX (mutually exclusive exon), A5 (alternative 5′ splice site), A3 (alternative 3′ splice site), RI (retained intron), AF (alternative first exon), and AL (alternative last exon).

Seven AS events were analyzed from the full-length transcripts of the FL-HG and FL-DG, including SE (skipped exon), MX (mutually exclusive exon), A5 (alternative 5′ splice site), A3 (alternative 3′ splice site), RI (retained intron), AF (alternative first exon), and AL (alternative last exon) (Figure 2A). These results showed that the total number of AS was 6619 in the FL-HG and 3795 in the FL-DG (Figure 2B). The number of AS events in the FL-HG was 2824 more than that in the FL-DG, and the number of AS events in each category was higher in the FL-HG than that in the FL-DG.

**Figure 3:**
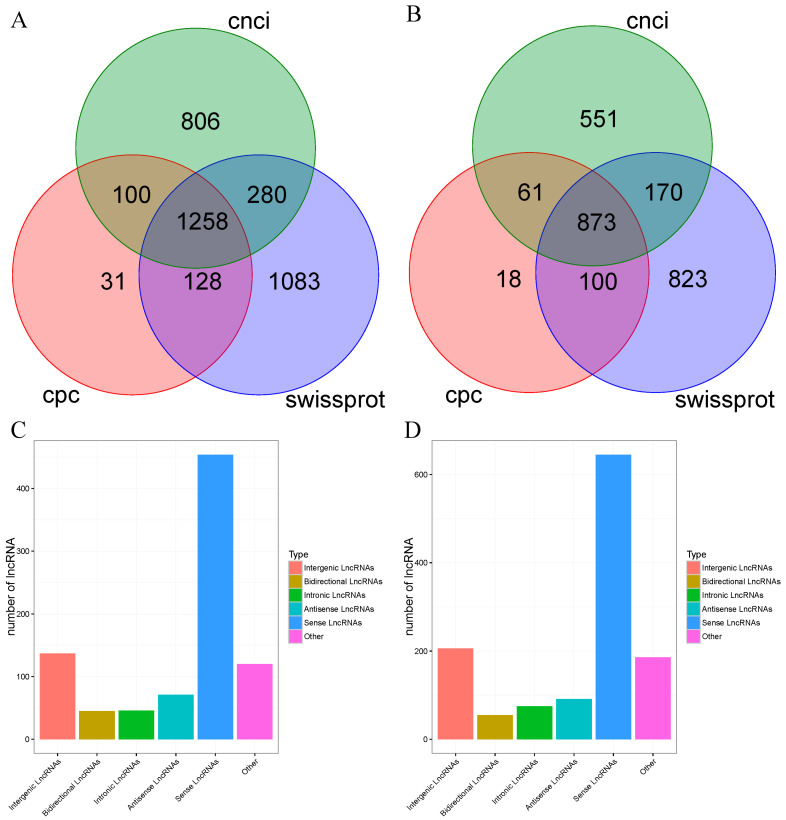
Long non-coding RNA (lncRNA) analysis based on the full-length transcripts. (**A**) Venn diagram of lncRNA prediction results in the FL-HG. (**B**) Venn diagram of lncRNA prediction results in the FL-DG. (**C**) Statistical graph of lncRNA classification results in the FL-HG. (**D**) Statistical graph of lncRNA classification results in the FL-DG.

In addition, lncRNA was also predicted using CNCI and CPC software, and the intersection of the three results predicted to be non-coding was used as reliable lncRNA results by comparing the Swiss-Prot protein database to find out the unannotated transcripts. The results showed that the number of lncRNA of the FL-HG predicted by CNCI, CPC, and Swiss-Prot was 2444, 1517, and 2749, respectively (Figure 3A). In addition, in the FL-DG, CNCI, CPC, and Swiss-Prot predicted 1655, 1052, and 1966 lncRNAs, respectively (Figure 3B). Subsequent analysis using the predicted lncRNAs in each database was also performed, and the results showed that there were 1258 and 873 lncRNAs in FL-HG and FL-DG, respectively. Based on the location of the lncRNAs in the genome, the results revealed that five types of lncRNAs were predicted in the FL-HG and FL-DG, including antisense, intronic, sense, bidirectional, and intergenic lncRNAs (Figure 3C,D).

### 3.4. Quality Assessment of Short-Read Sequencing Data

**Figure 4:**
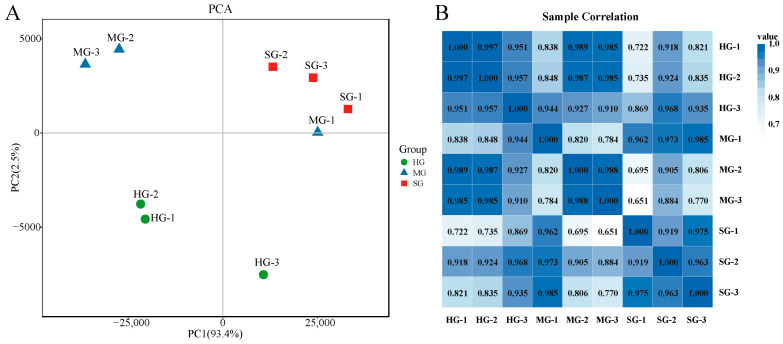
Sample relationship analysis. (**A**) Principal component analysis (PCA) of the genes in terms of variance across samples. (**B**) Sample correlation heat map.

We obtained a total of 363,378,170 raw reads, filtered to retain 362,027,932 clean reads (Table S1). The Q20 and Q30 of the clean reads ranged from 97.83 to 98.07% and from 93.95 to 94.43%, respectively. The genome total mapping rates were from 89.12% to 90.97% for each sample (Table S1). The results of principal component analysis (PCA) showed that PC1 and PC2 accounted for 93.4% and 2.5%, respectively (Figure 4A). Clustering heat map analysis based on Pearson’s correlation coefficient showed good reproducibility of gill samples across groups (Figure 4B).

### 3.5. Identification of Differentially Expressed Genes

**Figure 5:**
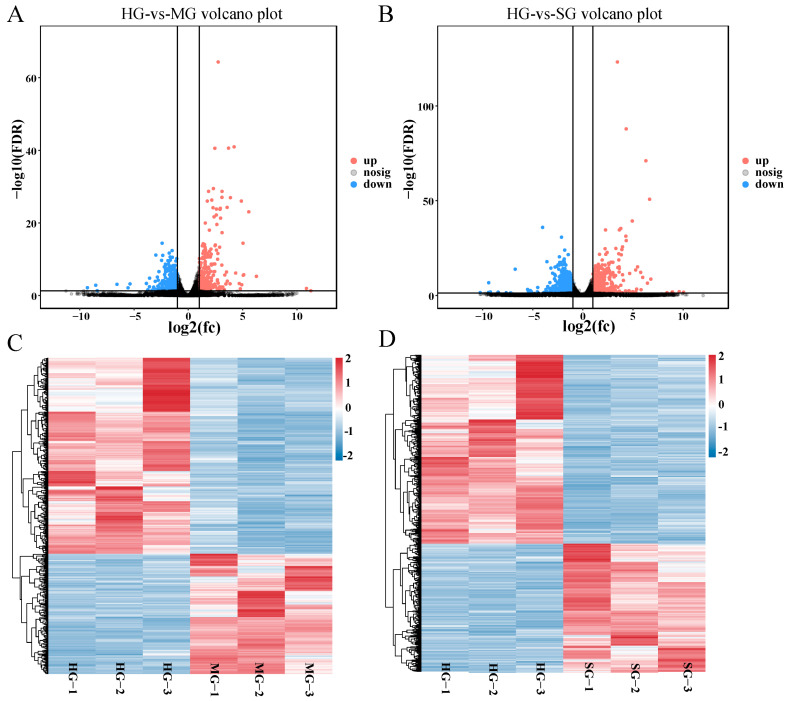
DEGs expression analysis. (**A**) Volcano plot of DEGs in the HG vs. MG group. (**B**) Volcano plot of DEGs in the HG vs. SG group. (**C**) Hierarchical clustering analysis DEGs in the HG vs. MG group. (**D**) Hierarchical clustering analysis of DEGs in the HG vs. SG group.

Initially, we performed pairwise comparisons between heathy and diseased fish groups to identify DEGs; however, only a small number of DEGs (54 upregulated and 94 downregulated) were detected in the MG vs. SG comparison. So, in the following analyses this comparison was excluded. Results showed that there were 687 DEGs in the HG vs. MG comparison (Figure 5A) (Table S2), including 260 upregulated genes and 427 downregulated genes in MG. The number of DEGs in the HG vs. SG comparison was 1535, including 617 upregulated genes and 918 downregulated genes in SG (Figure 5B) (Table S3). To visualize the expression profiles of the DEGs detected in healthy and infected fish, we plotted two heatmaps for the two comparisons in this study (Figure 5C,D). The transcriptome profiles of the healthy and infected samples were evidently similar across the sample samples within each group in the comparison.

### 3.6. Functional Analysis of DEGs

**Figure 6:**
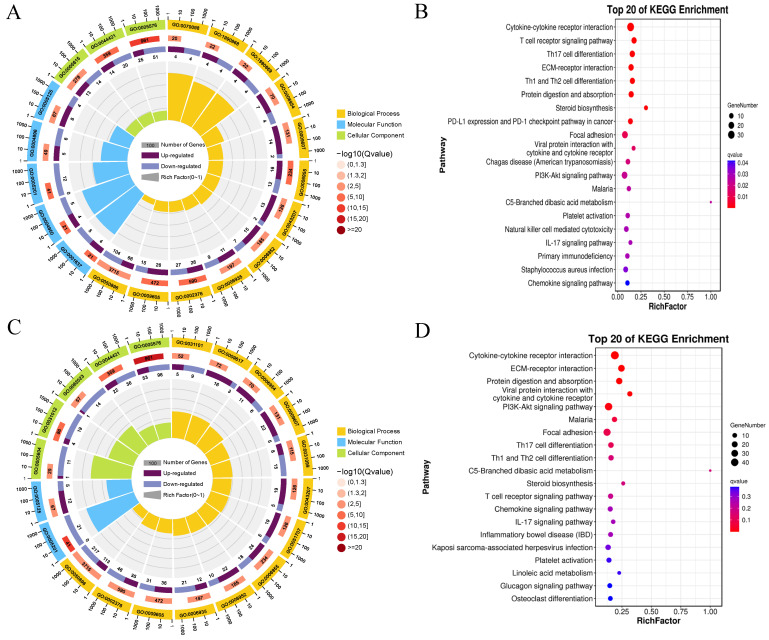
GO and KEGG function enrichment analysis of DEGs. (**A**) GO function enrichment analysis of the HG vs. MG group (top 20 enriched terms). (**B**) KEGG function enrichment analysis of the HG vs. MG group (20 enriched terms). (**C**) GO function enrichment analysis of the HG vs. SG group (top 20 enriched terms). (**D**) KEGG function enrichment analysis of the HG vs. SG group (top 20 enriched terms).

All DEGs were classified into three GO categories: biological process, molecular function, and cellular component. The top 20 GO terms enriched with DEGs of the HG vs. MG and HG vs. SG groups are shown in Figure 6A,C, respectively. In the HG vs. MG comparison, the first three cellular component categories were extracellular region (GO:0005576), extracellular region part (GO:0044421), and extracellular space (GO:0005615). The first three major biological processes were immune system process (GO:0002376), immune response (GO:0006955), and response to external stimulus (GO:0009605). The top three molecular functions were the extracellular matrix structural constituent (GO:0005201), G protein-coupled chemoattractant receptor activity (GO:0001637), and chemokine receptor activity (GO:0004950) (Figure 6A). In the HG vs. SG comparison, the first three cellular component categories represented were extracellular region (GO:0005576), extracellular region part (GO:0044421), and extracellular matrix (GO:0031012). The top two molecular functions were extracellular matrix structural constituent (GO:0005201) and cytokine activity (GO:0005125). The first three biological processes were the immune system process (GO:0002376), immune response (GO:0006955), and response to external stimulus (GO:0009605) (Figure 6C).

In the HG vs. MG comparison, the first five KEGG categories were cytokine–cytokine receptor interaction (ko04060), the T-cell receptor signaling pathway (ko04660), Th17 cell differentiation (ko04659), ECM–receptor interaction (ko04512), and Th1 and Th2 cell differentiation (ko04658) (Figure 6B). In the HG vs. SG comparison, the first five categories were cytokine–cytokine receptor interaction (ko04060), ECM–receptor interaction (ko04512), protein digestion and absorption (ko04974), viral protein interaction with cytokine and cytokine receptor (ko04061), and the PI3K-Akt signaling pathway (ko04151) (Figure 6D). 

### 3.7. GSEA Enrichment Analysis

**Figure 7:**
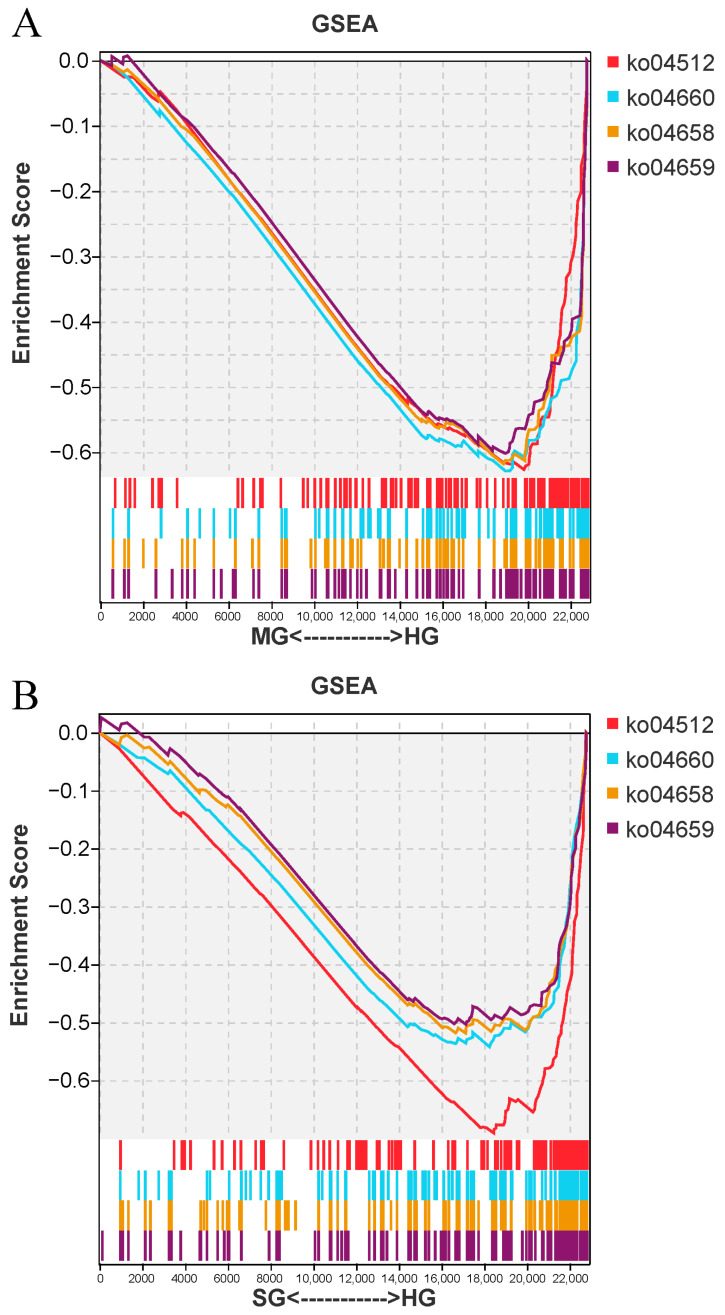
Pathway based GSEA results of the HG vs. MG (**A**) and HG vs. SG (**B**) groups.

GSEA enrichment analysis was conducted to clarify the functional gene sets closely related to *C. irritans* infection. Notably, we found that four immune-related enrichment gene sets were all downregulated in two groups of infected fish, including ko04512 (ECM-receptor interaction), ko04660 (T-cell receptor signaling pathway), ko04658 (Th1 and Th2 cell differentiation), and ko04659 (Th17 cell differentiation) (Figure 7).

### 3.8. Immune-Related DEGs and Protein–Protein Interaction Networks

**Figure 8:**
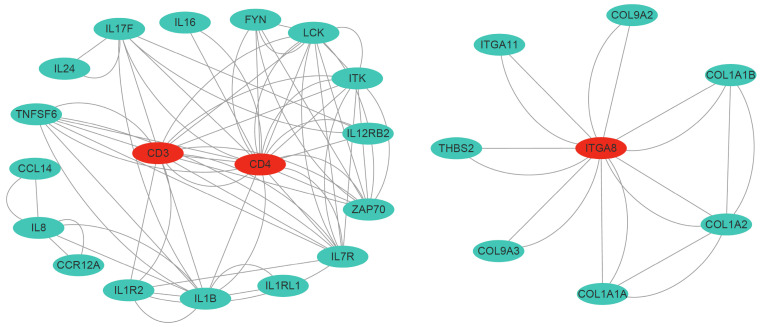
PPI networks of selected key DEGs. The red color indicates the hub genes.

We found that the expression levels of ECM–receptor interaction-related genes (THBS2, LAMA5, ITGA8, and COL1A1A) were downregulated in infected fish (Table 2). Many cytokines (e.g., CCR12A, CCL14, IL1R2, and TNFRSF2) were differentially expressed in healthy and infected fish. In addition, we identified many genes related to T-cell differentiation that were downregulated in infected fish, such as CD4, FYN, ZAP70, and LCK. Overall, the vast majority of the selected DEGs were distinctly downregulated in infected fish. To reveal the relationship of the immune-related DEGs, a PPI network was constructed (Figure 8). Some nodes with many edges were considered as hub genes (CD3, CD4, and ITGA8), which may play important roles in the immune response to parasite infection.

### 3.9. Overlapping Genes between DEGs and Candidate Genes in QTLs

In our previous study, two putative major QTLs associated with *C. irritans* resistance were identified via GWAS, and a total of 32 candidate genes were annotated. By comparing these genes with DEGs from the HG vs. MG and HG vs. SG comparison groups, five (NDUFB6, PRELID1, SMOX, SLC25A4, and DENND1B) of the 32 genes were overlapped genes (Table 3).

## 4. Discussion

This study highlights key adaptive immune responses in fugu when infected with *C. irritans*, revealing potential molecular mechanisms through which the host responds and defends against the invading parasites, and the identified pathways could potentially target the parasite as it seeks to evade the host’s defenses during infection and multiplication. Here, we determined the full-length transcriptome of the gills of healthy and infected fish. By comparing the reference genome, 47,307 and 34,413 full-length transcripts were obtained from healthy and infected fish, respectively. Furthermore, AS events and lncRNAs were predicted to deepen our understanding of the complexity of the fugu transcriptome. We found that the likelihood of AS events was reduced after infection with *C. irritans*, the number of AS events was 6619 and 3795 in healthy and infected fish, respectively, and all seven AS types were more abundant in healthy than in infected fish. Meanwhile, the number of lncRNAs was lower in infected fish (873) than in healthy fish (1258). Thus, changes in the type and number of AS events and lncRNAs after infection with *C. irritans* suggest that they may be involved in the regulation of immune mechanisms against *C. irritans* in fugu. However, whether these observed differences are specific to *C. irritans* infection or common to other pathogenic or parasitic infections remains to be further investigated.

To gain more insights into the immune response behind *C. irritans* infection, we analyzed the gill transcriptome profile and identified DEGs in fugu. Here, we detected more DEGs in heavily infected fish (1535) compared to moderately infected fish (687), relative to the healthy fish. This might be attributed to the fact that severely infected fish need a stronger immune response to combat this level of immune stimulus infection, which subsequently results into a higher number of differently stimulated genes. These DEGs will provide valuable information for future studies regarding *C. irritans* host–parasite interactions in fugu. Functional enrichment analysis further explored the functional involvement of the DEGs in host immune responses. Genes involved in ECM-receptor interaction, T-cell reporter signaling pathway, Th1 and Th2 cell differentiation, and Th17 cell differentiation pathways were significantly enriched in the identified DEGs and were expressed at low levels in infected fish relative to the healthy ones. Furthermore, the GSEA results indicated that these pathways were inhibited (downregulated) in infected fish as compared to healthy fish. These results reveal that the immune response in the gills of fugu may be suppressed after infection with *C. irritans* in fugu.

The host extracellular matrix (ECM) plays an important role in the immune responses to pathogen infection in multicellular organisms. Pathogens and host immune cells can undergo complex interactions in the context of the ECM [37]. Pathogens such as parasites can adhere to, degrade, or alter the ECM components of the host to make cellular invasion more favorable [38,39,40]. It has been shown that 52 significant DEGs are enriched in the ECM–receptor interaction pathway in the gills of the silver pomfret (*Pampus argenteus*) after *Amyloodinium ocellatum* infection [41]. Additionally, the expression levels of extracellular molecules, including tenascin and thrombospondin, were upregulated in the head kidney of *P. argenteus* after infection with *C. irritans*, whereas the expression levels of collagen and perlecan genes were downregulated [42]. In the current study, we identified some key DE genes associated with ECM–receptor interactions, including thrombospondin, laminin, integrins, and collagen. Interestingly, all these DE genes were downregulated in infected fish relative to healthy fish, suggesting that *C. irritans* infection may potentially antagonize gill homeostasis and the extracellular matrix in the gill tissue. Integrins have been observed to enhance the interactions between antigen-presenting cells and T lymphocytes by interacting with the ECM [43]. As a hub gene, ITGA8 was significantly downregulated in infected fish. Though studies of the involvement of integrins in immune defense in fugu are scarce, limited studies suggest that integrins may be potential biomarkers for predicting immune responses to disease in fish undergoing inflammation [16]. Thus, disruption of intercellular communication and structure in infected fugu is likely to impair the cellular immune response, which in turn contributes to survival and pathogenicity of *C. irritans*.

Inflammation is an immunological protective response in organisms that plays a key role in pathogen clearance and healing of injured tissues [44]. Previous studies have reported that the inflammatory response of *L. crocea* may be suppressed during *C. irritans* infection [14]. Almost all aspects of immunity and inflammation are regulated by cytokines [45]. It has been reported that cytokines are associated with host defense against *C. irritans* infection in *L. crocea* [13]. In the present study, DE genes from both comparison groups were significantly enriched in the cytokine–cytokine receptor interaction pathway. Differentially expressed cytokines transmit inflammatory signals in different ways and can alter the ability of phagocytes to destroy pathogens [46]. In our study, we found that a large number of cytokines (chemokines, interleukins, and tumor necrosis factor family genes) were downregulated in infected fish. Taken together, these findings suggest that the inflammatory response is critical for *T. rubripes* resistance to *C. irritans* infection. 

In this study, many downregulated DEGs were involved in adaptive immune pathways, such as the T-cell receptor signaling pathway, Th1 and Th2 cell differentiation, and Th17 cell differentiation, suggesting that infection with *C. irritans* predominantly affects adaptive immune responses. Adaptive immunity, which relies on the function of T cells, B cells, and their sub-populations to directly destroy infected cells, is a critical aspect of vertebrate evolution [47]. T lymphocytes produce cytokines that mediate inflammation [48]. The initiation of the T-cell receptor (TCR) signaling pathway depends on the interaction of membrane TCR co-receptors and a range of cytoplasmic protein tyrosine kinases [49,50]. In the present study, two TCR co-receptors (CD3 and CD4) and three kinases (Lck, Fyn, and ZAP70) were downregulated in infected fish gill tissue, suggesting that these T cells may be suppressed in gills. CD4, a potent cell-surface molecule expressed by T-helper cells, may therefore serve as a potential molecular indicator gene of *C. irritans* infection in fugu [51]. This suppression of T cell-related genes supports the overall conclusion that *C. irritans* infection suppresses the immune response in fugu.

In addition, SMOX and DENND1B are two overlapping genes identified by RNA-seq and GWAS as being associated with *C. irritans* infection in fugu. SMOX is one of the key enzymes in arginine metabolism, capable of decomposing polyamine and spermine [52]. Spermine has been reported to increase IL-10 synthesis and inhibit the production of the p40 subunit of IL-12 and interferon-γ in macrophages [53]. It has been shown that dietary supplementation of arginine suppressed certain immune mechanisms in European sea bass (*Dicentrarchus labrax*), leading to increased susceptibility to disease [54]. Therefore, SMOX may play an important role in the immune mechanism of *T. rubripes*. DENND1B has been reported to modulate T-cell receptor signaling and is involved in the immune response and the development of various diseases [55]. Additionally, DENND1B balances inflammation and apoptosis by acting as a potential regulator of NFκB [56]. In the present study, DENND1B was significantly downregulated in severely infected fish, suggesting that inflammatory responses may be suppressed. Although their exact role in the immune mechanisms of *C. irritans* infection is unknown, further studies of their function may shed light on their immunological roles in fugu. The downregulation of these genes further supports the notion that *C. irritans* infection leads to a suppressed immune response.

Many parasites have developed various strategies to avoid detection, suppress immunity, and deviate immune attack mechanisms [57,58]. For example, during helminth infections, worms inhibit the expression of TLRs, thereby preventing the development of Th1/Th17 cells [59]. Trypanosomes continually change their surface antigens after infecting a host, thereby evading host immune clearance [60]. Our findings suggest that *C. irritans* can adopt a potential immune evasion strategy by suppressing the immune response processes, which may be one of the reasons for the large-scale outbreaks of cryptocaryoniasis in fugu aquaculture. However, the underlying mechanisms of fish interactions with *C. irritans* is not clear, with some studies showing immune activation in fish [11,15,61] and others reporting immune suppression in fish [14,42]. Notably, these studies differ in terms of the host species studied, the target tissues, and the experimental layout. Therefore, the exact mechanism of adaptive immune evasion mediated by *C. irritans* requires further studies utilizing some of the most genomic technologies such as single-cell genomics and single-cell epigenomics, and considering multiple immune tissues in the body.

## 5. Conclusions

In summary, this study constructed full-length transcripts and analyzed their structure and function for the first time in fugu, which further enriched the gene resources of this species. In addition, the immune response of fugu under *C. irritans* infection was investigated via short-read RNA sequencing of the gill tissue. Our analyses revealed multiple immune-related pathways (i.e., ECM–receptor interaction, T-cell reporter signaling pathway, Th1 and Th2 cell differentiation, and Th17 cell differentiation pathway) that are involved in the response to *C. irritans* infection in fugu, and these pathways potentially inhibited upon parasite infection. Interestingly, we identified five key genes (NDUFB6, PRELID1, SMOX, SLC25A4, and DENND1B) that are likely to be associated with *C. irritans* resistance according to the combined comparison between DEGs and preciously identified candidate genes in QTL regions associated with resistance of the parasite in the same fugu population. Our findings contribute to an in-depth understanding of host immune response to *C. irritans* infection, as well as the pathogenesis mechanism of the parasite infection, with the potential to be utilized to improve fugu fish welfare under aquaculture systems.

## Figures and Tables

**Table 1:** Overview of the result of PacBio Iso-seq data.

Subjects	Data	Number	
		FL-HG	FL-DG
Subreads	Total base	58,401,291,009	44,613,937,642
	Subreads number	26,318,483	21,660,262
	Average length	2219	2059
	N50	2395	2211
Number of CCS	Number of CCS reads	786,806	609,313
	FLNC reads number	704,548 (89.55%)	505,811 (83.01%)
	Mean length of FLNC	2410	2130
Number of isoforms	HQ isoform number	47,307	34,413
HQ isoform mapped to genome	Unique mapped (%)	35,931 (75.95%)	25,614 (74.43%)
	Multiple mapped (%)	385 (0.81%)	271 (0.79%)
	Unmapped (%)	10,991 (23.23%)	8528 (24.78%)

**Table 2:** The key candidate DEGs shared in two comparison groups.

ID	Description	Abbreviation	Log_2_ Fold Change	
			HG vs. MG	HG vs. SG
Chemokines and Chemokine Receptors				
ENSTRUG00000010223	C-C chemokine receptor type 12a	CCR12A	2.69	1.82
ENSTRUG00000019936	C-C chemokine receptor type 7	CCR7	−2.21	−1.59
ENSTRUG00000027740	C-C chemokine receptor type 9-like	CCR9A	−1.17	−1.26
ENSTRUG00000023885	C-C chemokine receptor type 9b	CCR9B	−2.09	−2.36
ENSTRUG00000029174	C-C motif chemokine 14-like	CCL14	−1.91	−2.07
ENSTRUG00000020129	Interleukin 1 receptor type 2	IL1R2	1.88	2.55
ENSTRUG00000016002	Interleukin 1 beta	IL1B	1.78	3.00
ENSTRUG00000023550	Interleukin 1 receptor type 1-like	IL1RL1	1.70	1.47
ENSTRUG00000026338	Interleukin 7 receptor	IL7R	−1.80	−2.00
ENSTRUG00000006788	Interleukin 8	IL8	2.58	3.78
ENSTRUG00000018372	Interleukin-12 receptor subunit beta-2-like	IL12RB2	−1.34	−1.19
ENSTRUG00000033166	Interleukin 16	IL16	−1.08	−1.03
ENSTRUG00000020732	Interleukin 17F-like	IL17F	−1.67	−1.53
ENSTRUG00000006725	Interleukin-24-like	IL24	2.67	2.84
ENSTRUG00000021770	Interleukin-6 receptor subunit beta-like	IL6RB	1.25	1.38
ENSTRUG00000017328	TNF receptor superfamily member 21	TNFRSF21	−1.45	−1.25
ENSTRUG00000021595	Tumor necrosis factor ligand superfamily member 6-like	TNFSF6	−1.43	−1.28
ECM-receptor interaction				
ENSTRUG00000031037	Thrombospondin 2	THBS2	−2.46	−2.78
ENSTRUG00000029497	Laminin subunit alpha 5	LAMA5	−1.91	−2.07
ENSTRUG00000013059	Integrin subunit alpha 8	ITGA8	−1.27	−1.40
ENSTRUG00000016742	Integrin subunit alpha 11	ITGA11	−1.24	−1.77
ENSTRUG00000013913	Collagen alpha-1a(I) chain-like	COL1A1A	−1.45	−2.18
ENSTRUG00000007520	Collagen alpha-1b(I) chain-like	COL1A1B	−1.31	−2.13
ENSTRUG00000015407	Collagen alpha-2(I) chain	COL1A2	−1.25	−2.03
ENSTRUG00000010345	Collagen alpha-1(II) chain	COL2A1	−2.81	−3.58
ENSTRUG00000006672	Collagen alpha-3(IX) chain	COL9A3	−1.79	−1.63
ENSTRUG00000015261	Collagen alpha-2(IX) chain	COL9A2	−1.57	−1.47
T/B cell activation and proliferation				
ENSTRUG00000010193	Phosphatidylinositol-4,5-bisphosphate 3-kinase catalytic subunit delta	PIK3CD	−1.01	−1.09
ENSTRUG00000003538	FYN proto-oncogene, Src family tyrosine kinase	FYN	−1.06	−1.16
ENSTRUG00000006051	Zeta chain of T cell receptor associated protein kinase 70	ZAP70	−1.39	−1.45
ENSTRUG00000004669	LCK proto-oncogene, Src family tyrosine kinase	LCK	−1.30	−1.48
ENSTRUG00000003789	CD3 gamma/delta	CD3	−1.48	−1.28
ENSTRUG00000010854	T-cell surface glycoprotein CD4	CD4	−1.76	−2.34
ENSTRUG00000004405	IL2-inducible T-cell kinase	ITK	−1.23	−1.39
ENSTRUG00000018363	Nuclear factor of activated T-cells, cytoplasmic 2-like	NFATC2	−1.16	−1.51
ENSTRUG00000021189	Interferon regulatory factor 4-like	IRF4A	−1.20	−1.23
ENSTRUG00000022517	Forkhead box P3b	FOXP3B	−2.10	−2.07
ENSTRUG00000018285	Transcription factor Maf-like	MAF	−1.28	−1.04

**Table 3:** Candidate genes shared between DEGs, and candidate genes identified via GWAS.

ID	Description	Symbol	Log_2_ Fold Change	
			HG vs. MG	HG vs. SG
ENSTRUG00000003741	NADH:ubiquinone oxidoreductase subunit B6	NDUFB6	-	1.06
ENSTRUG00000005382	PRELI domain containing 1	PRELID1	-	1.07
ENSTRUG00000001507	Spermine oxidase	SMOX	1.74	1.95
ENSTRUG00000005070	Solute carrier family 25 member 40	SLC25A40	-	1.24
ENSTRUG00000017613	DENN domain-containing protein 1B-like	DENND1B	-	−1.16

## Data Availability

All the sequencing data used in this study were deposited at the China National Center for Bioinformation (https://www.cncb.ac.cn/, accessed on 4 June 2024) with accession number CRA016702.

## Supplementary Materials

The following supporting information can be downloaded at: https://www.mdpi.com/article/10.3390/ani14142058/s1, Table S1: Information of RNA-seq data; Table S2: DEGs in the HG vs. MG group. Table S3: DEGs in the HG vs. SG group.
